# Characteristics and outcomes of non-Hodgkin’s lymphoma patients with leptomeningeal metastases

**DOI:** 10.1007/s10147-018-1268-5

**Published:** 2018-03-20

**Authors:** Xiangrui Meng, Jingwei Yu, Qian Fan, Lanfang Li, Wei Li, Zheng Song, Xianming Liu, Yanyang Jiang, Ming Gao, Huilai Zhang

**Affiliations:** 1 Department of Lymphoma, Tianjin Medical University Cancer Institute and Hospital, National Clinical Research Center for Cancer, Key Laboratory of Cancer Prevention and Therapy, Tianjin, 300060 People’s Republic of China; 2 Tianjin’s Clinical Research Center for Cancer, Tianjin, People’s Republic of China; 3 Department of Social Science, Florida State University, Tallahassee, FL 32306 USA; 4 Department of Thyroid and Neck Tumor, Tianjin Medical University Cancer Institute and Hospital, National Clinical Research Center for Cancer, Key Laboratory of Cancer Prevention and Therapy, Tianjin, 300060 People’s Republic of China

**Keywords:** Non-Hodgkin’s lymphoma, Leptomeningeal metastases, Characteristics

## Abstract

**Background:**

Leptomeningeal metastasis is an uncommon but devastating complication. The incidence of non-Hodgkin’s lymphoma has been increasing in recent decades, due to the poor central nervous system penetration of drugs and the prolonged overall survival of patients, leptomeningeal metastases has gradually increased over time. Patients with leptomeningeal metastases have short survival durations and poor quality of life; there are few studies about non-Hodgkin’s lymphoma with leptomeningeal metastases. We investigated characteristics and outcomes of non-Hodgkin’s lymphoma patients with leptomeningeal metastases.

**Methods:**

This study included 27 non-Hodgkin’s lymphoma patients with leptomeningeal metastases diagnosed at Tianjin Medical University Cancer Institute and Hospital between 2013 and 2016. Statistical analysis was performed to investigate the overall survival of non-Hodgkin’s lymphoma with leptomeningeal metastases.

**Results:**

Diffuse large B cell lymphoma was the most common cancer subtype (21/27, 78%), and more than half of the patients showed extranodal involvement (18/27, 67%). Survival analysis has shown extranodal involvement (*P* = 0.0205), International Prognostic Index (*P* = 0.0112), performance status (*P* < 0.0001), parenchymal involvement (*P* = 0.0330) and received radiotherapy (*P* = 0.0056) were predictive factors of prognosis for these patients with leptomeningeal metastases. Cox regression analysis has shown patients with concurrent parenchymal involvement and received radiotherapy are correlated with good prognosis.

**Conclusions:**

Given the small number of patients who were included, this study exhibited limitations with respect to analytical power and the random selection of patients. Nevertheless, this investigation revealed characteristics of non-Hodgkin’s lymphoma patients with leptomeningeal metastases and suggested that such patients could benefit from multimodal therapy.

**Electronic supplementary material:**

The online version of this article (10.1007/s10147-018-1268-5) contains supplementary material, which is available to authorized users.

## Introduction

Non-Hodgkin’s lymphoma (NHL) is a highly heterogeneous group of hematological tumors with many subtypes that range from indolent to highly aggressive. With the clinical application of chemotherapy and targeted agents, overall survival among NHL patients has improved greatly in recent decades. Due to the different histopathological subtypes of lymphoma, the risk of central nervous system (CNS) recurrence ranged from 2.8 to 24.4% [1, 2]. Some clinical studies of diffuse large B cell lymphoma (DLBCL) [3, 4], addition of rituximab to chemotherapy reduced the incidence of CNS disease, but the benefit from adding rituximab to CHOP is limited, CNS relapse still a clinical challenge. CNS involvement can include leptomeningeal involvement and parenchymal brain lesions, the former of which is more frequent [2, 5–9]. Leptomeningeal metastases (LM) is a devastating condition that refers to involvement of the leptomeninges or cerebrospinal fluid (CSF) in the context of any malignant cancer. LM results from the multifocal seeding of the leptomeninges by cancer cells. For NHL, the frequency of LM increases with the clinical aggressiveness of the tumor. Neurological symptoms are typical manifestations of LM, which severely affects quality of life.

Because LM characteristically has low incidence and an occult onset and is difficult to diagnose, patients with LM have short survival durations, there are few studies about NHL patients with LM. The treatment goal for these patients is to improve the quality of life by repairing neurological deterioration. However, there are no randomized studies to define the optimal management and treatment for LM. A combination of intrathecal chemotherapy, systemic chemotherapy and whole brain radiotherapy is recommended by oncologists, but there lack of prospective clinical trials to support. There are instances of LM patients with prolonged survival, so identified LM patients with various risk factors into subgroups is critical for prognosis.

Here, we report experiences with NHL patients with LM at a single institution, Tianjin Medical University Cancer Institute and Hospital, with the objective of characterizing LM and suggesting a practical approach for managing this condition.

## Patients and methods

### Patients

From 2013 to 2016, 2784 patients were diagnosed with NHL at Tianjin Medical University Cancer Institute and Hospital, of which 27 patients were diagnosed as LM (as shown in supplementary Tables 1 & 2). Institutional review board approval to retrospectively review these patients’ records and utilize these records to report outcomes was obtained. The criteria for LM diagnosis were as follows: CSF positive for tumor cells; magnetic resonance imaging (MRI) with supportive clinical findings; and/or signs and symptoms with CSF suggestive of LM. Treatment principle was in accordance with National Comprehensive Cancer Network (NCCN) guidelines for Central Nervous System Cancers 2011 Version 2. Intrathecal (IT) therapy was the initial treatment for all patients. In particular, patients received intra-CSF treatment with 10 mg methotrexate (MTX) or 50 mg cytarabine (Ara-C) and concurrent dexamethasone (10 mg). According to NCCN guidelines for Central Nervous System Cancers, CSF cytology positive treated with induction intra-CSF chemotherapy, treatment was administered 2–3 times per week. When CSF cytology was negative, continue to give induction intra-CSF chemotherapy for 1 month, and then give maintenance treatment, which administered every 4 weeks until progression, toxicity, or patient-determined discontinuance occurred. The IT treatment schedule was adjusted based on toxicity. Systemic treatment depended on systemic disease(s) and prior treatment history. The median patient follow-up time was 25.4 months (range 2.2–43.3 months).

### Statistical analysis

Statistical analyses were performed to examine outcomes for NHL patients who had developed LM. Patients’ overall survival times were summarized using the Kaplan–Meier method. Prism 6.0 Software (GraphPad, Inc., La Jolla, CA, USA) was used for statistical analyses. Multivariate modeling of survival was performed using cox regression SPSS version 22.2.0 (IBM, Armonk, NY, USA).

## Results

### Clinical characteristics

The median age of the 27 patients was 52 years (range 19–64 years), and 7 patients were older than 60 years of age. The patient cohort included 18 males (67%). The most frequent histologic subtype of NHL was diffuse large B-cell lymphoma (DLBCL) (21 patients, 78%), followed by peripheral T cell lymphoma, Burkitt’s lymphoma, and mantle cell lymphoma. At the time of diagnosis, 6 patients presented with B symptoms, 7 patients presented with LM, and one-third of the patients had advanced disease (with 8 patients (30%) diagnosed with stage IV NHL). In total, 18 patients (67%) exhibited extranodal involvement. The most common extranodal sites were the testes and nasal cavity (4 patients each), followed by the breasts, CNS, and appendix. Initially, 30% of the patients had intermediate or high International Prognostic Index (IPI) scores. The patients’ main demographic and clinical characteristics are listed in Table 1.

**Table 1:** Characteristics of 27 NHL patients diagnosed with LM

Patients’ characteristics	Value
Age (years)	
Mean	48 (19–64)
Median	52 (19–64)
< 60	20 (74%)
> 60	7 (26%)
Gender	
Male	18 (67%)
Female	9 (33%)
Histopathology	
DLBCL	21 (78%)
PTL	3 (11%)
Burkitt’s	2 (7%)
MCL	1 (4%)
Stage	
I–II	17 (63.0%)
III–IV	10 (37.0%)
B symptom	
No	21 (78%)
Yes	6 (22%)
Extranodal involvement	
No	9 (33%)
Yes	18 (67%)
IPI score	
0–1 (low)	19 (70%)
2–3 (intermediate)	6 (22%)
4–5 (high)	2 (8%)
KPS	
0–50	4 (15%)
60–80	21 (78%)
90–100	2 (7%)
LM at primary diagnosis	7 (26%)

*LM* leptomeningeal metastases, *IPI score* International Prognostic Index score, *KPS* Karnofsky performance status

IT therapy was administered in all cases; 7 of the 27 patients presented with LM at the time of diagnosis, and the remaining 20 patients presented with LM at relapse. All patients had malignant cells in the CSF, positive MRI results were obtained for 20 patients, and 12 of the 27 patients underwent flow cytometry, which produced positive results in each case. Patients’ neurological symptoms included headache, dizziness, limb weakness, nausea, vomiting, diplopia, blurred vision, aphasia, hearing impairment, hypogeusia, limb paresthesia, facial palsy, and ptosis (Table 2).

**Table 2:** Neurological symptoms of the 27 NHL with LM

Neurological symptoms	Value
Dizziness and headache	20 (74%)
Limb weakness	15 (56%)
Nausea and vomiting	13 (48%)
Diplopia or blurred vision	7 (26%)
Aphasia	3 (11%)
Hearing impairment	2 (7%)
Hypogeusia	2 (7%)
Limb paresthesia	2 (7%)
Facial paralysis	1 (4%)
Blepharoptosis	1 (4%)
Asymptomatic	2 (7%)

As indicated in Table 3, the most common IT therapy was MTX. The IT therapy administration schedule used in most cases (*n* = 19) was 2–3 treatments per week during the induction phase followed by weekly treatment. Lumbar puncture was the main route for IT administration (18 out of the 27 patients), and an Ommaya reservoir was used for 9 patients. Whole brain radiotherapy was administered to 4 patients, and cranial and spinal radiotherapy was administered to 6 patients.

**Table 3:** Treatment in the 27 NHL patients with LM

Intrathecal and other CNS-directed therapies	Value
Drugs of intrathecal therapy	
MTX	27 (100%)
Ara-C	18 (67%)
Route of administration of i.t. therapy	
Lumbar puncture	18 (67%)
Intraventricular (Ommaya reservoir)	9 (33%)
Frequency of administration of i.t. therapy in induction phase	
2–3 times per week	19 (70%)
Weekly	8 (30%)
Radiotherapy	
Whole brain radiotherapy	4 (15%)
Cranial and spinal radiotherapy	6 (22%)

*i.t. therapy* intrathecal therapy

### Treatment and survival

**Fig. 1:**
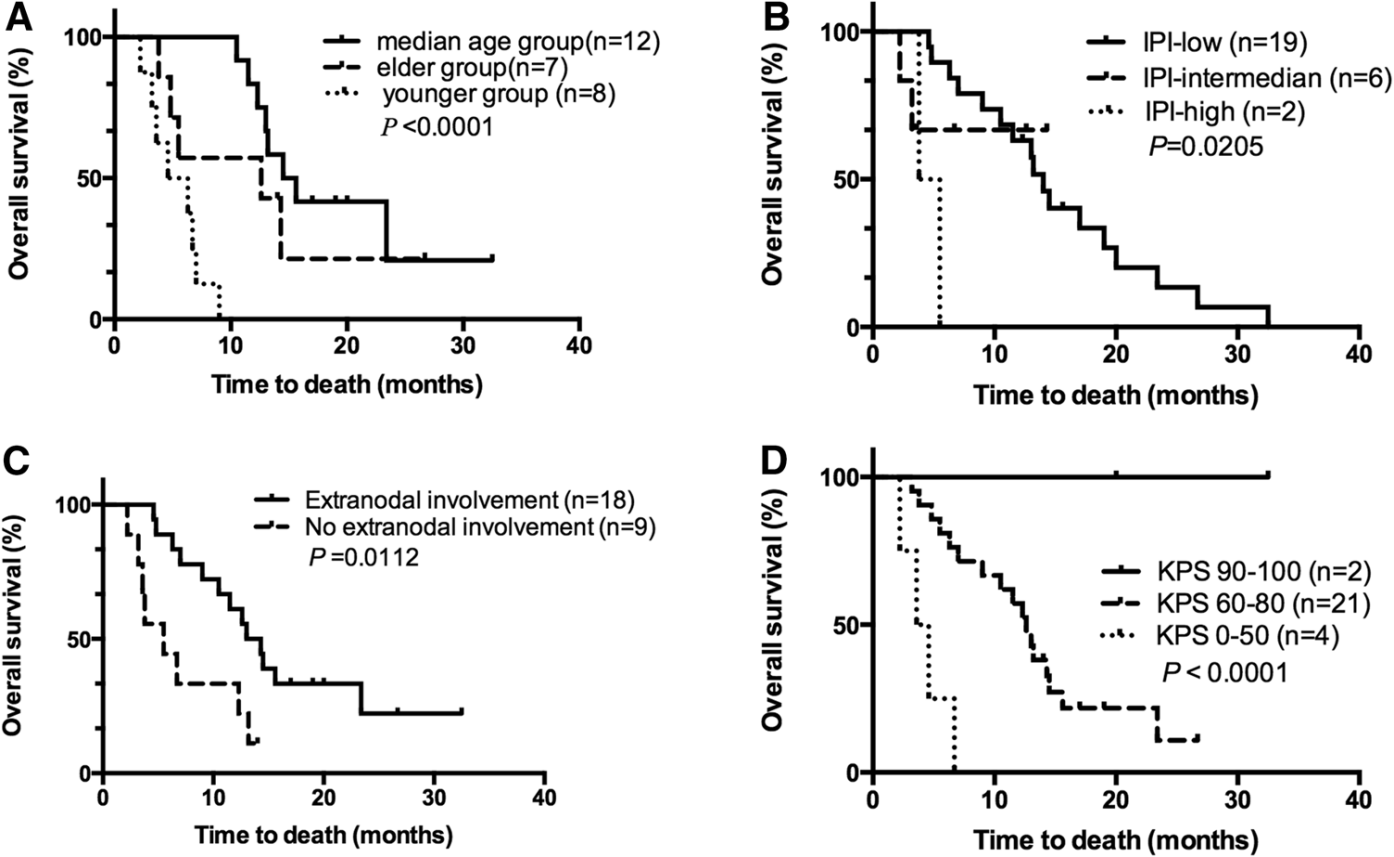
**a** OS of NHL patients with LM by different age group. **b** OS of NHL patients with LM with or without extranodal involvement. **c** OS of NHL patients with LM according to IPI scores. **d** OS of NHL patients with LM by KPS scores

**Fig. 2:**
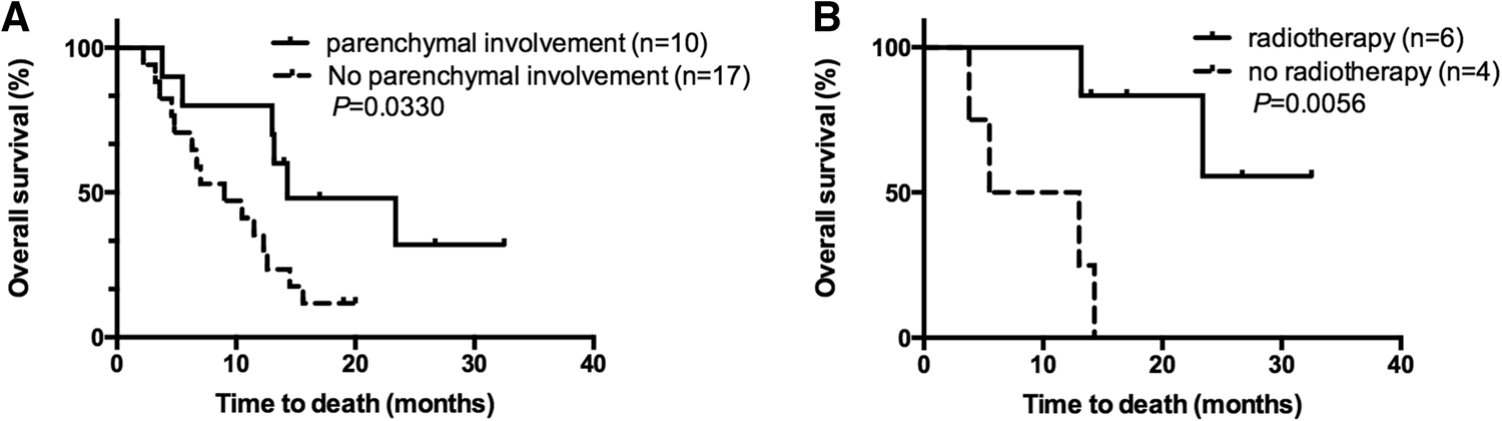
**a** OS of NHL patients with LM with or without parenchymal involvement. **b** OS of NHL patients with LM complicated with parenchymal involvement received or not received radiotherapy

**Fig. 3:**
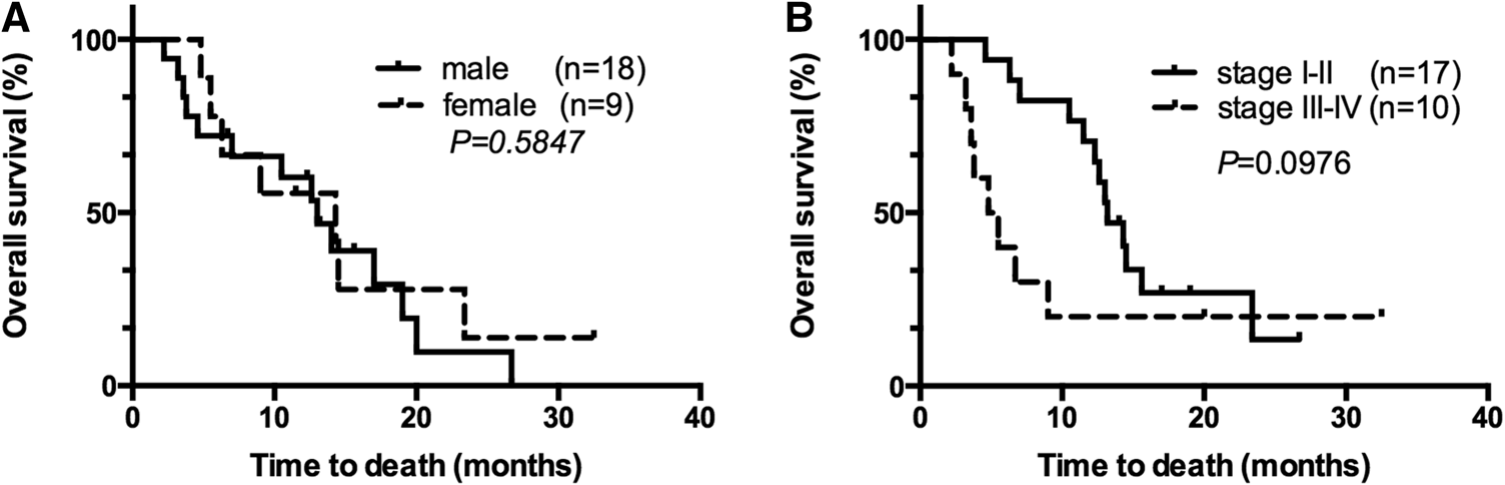
**a** OS of NHL patients with LM by gender. **b** OS of NHL patients with LM by different stage

The patients were divided into three groups by age (the younger group, 18–30 years; the median age group, 31–60 years; and the elder group, > 60 years). Survival analysis indicated that the younger group patients had the worst prognosis, followed by the elder group patients; the median age group patients had the best prognosis (*P* < 0.0001, Fig. 1a). Patients with extranodal involvement exhibited a worse prognosis than patients without extranodal involvement (*P* = 0.0112, Fig. 1b). High IPI score was associated with poor prognosis (*P* = 0.0205, Fig. 1c). Initial Karnofsky Performance Status (KPS) at LM diagnosis was better for long-term survivors than for patients with poor survival (*P *< 0.0001, Fig. 1d). There were 10 patients with parenchymal involvement, and these patients had better prognoses than the remaining subjects (*P* = 0.0330, Fig. 2a). Of the 10 complicated with parenchymal involvement patients, 6 patients received radiotherapy had a longer survival the 4 patients without radiotherapy (*P* = 0.0056, Fig. 2b). Gender and initial stage did not have significant effects on prognosis (*P* = 0.5847 and *P* = 0.0976, respectively, Fig. 3a, b). Cox regression analysis has shown that patients with concurrent parenchymal involvement and who received radiotherapy are correlated with good prognosis (as shown in supplementary Table 3).

## Discussion

The incidences of CNS relapse reported in the literature [2, 10–13] range from 1.6 to 5%, and the incidence of LM is even lower. Thus, LM is an uncommon but devastating complication for NHL patients, although such patients experience prolonged survival due to improved local and systemic therapies. LM also presents a challenge for oncologists and hematologists.

### Clinical presentation and characteristics

At presentation, 5 out of the 7 NHL patients with LM at diagnosis reported neurological symptoms. Only two patients were diagnosed with LM via systemic examination, and both of these patients exhibited relatively long-term survival. Multiple studies [12, 14] have found that CNS recurrences occur early in NHL cases and that most patients who experienced a CNS event presented with CNS manifestations during their initial chemotherapy. For example, the median times to CNS recurrence reported by Bernstein et al. [14] and Besien et al. [12] were 5.4 and 6 months, respectively. In our study, the median time to LM diagnosis was 9.4 months from initial NHL diagnosis. This result indicates that LM may occur early, even during chemotherapy. These data suggest that certain patients had subclinical CNS disease at diagnosis and that early diagnosis may improve survival.

In our study, survival analysis showed that the younger group patients had the worst prognosis, followed by the elder group patients. Median age group patients showed the best prognosis. It appears likely that the younger group patients with LM had highly aggressive disease that progressed rapidly and that the elder group patients were less tolerant of systemic treatment. These findings suggest that age at LM diagnosis and treatment modality may impact survival. Initial KPS scores at LM diagnosis were better for patients with prolonged survival than for patients with poor survival. In part, better KPS score reflected a lower CNS disease burden; this conclusion is further supported by the fact that none of the long-surviving patients presented with radiographically bulky disease or LM-related hydrocephalus. This lower disease burden allowed a larger range of systemic therapies to be administered to these long-surviving patients with NHL-related LM. Studies [6, 14–16] have demonstrated that patients with more than one extranodal site have significantly higher rates of CNS relapse. Extranodal involvement is a risk factor that multiple studies have identified as predictive of CNS relapse [2, 10–12, 14]. In this study, extranodal site involvement was also a major risk factor for poor prognosis, and there were no cases with bone marrow involvement.

IPI score was reported to be a highly predictive factor for overall prognosis in case series [17]. IPI score is also predictive of the risk of CNS relapse [14]. In our study, patients with high IPI scores had poor prognoses, suggesting that high IPI score may be an additional predictive factor for prognosis for patients with LM.

### Treatment

The determination of which LM patients to treat is challenging, and there exist few guidelines describing how prolonged survival can be achieved. Treatment for LM commonly involves a combination of IT and systemic therapy. Four commonly used drugs are liposomal Ara-C, Ara-C, MTX and thiotepa, all of which result in similar OS. In our center, we customarily utilize MTX and Ara-C. Clinical trials of new intra-CSF agents such as rituximab for the treatment of CD20-positive B-cell lymphoma are being performed, and the results [18–20] suggest that rituximab is feasible and highly effective for IT treatment. A large-scale prospective clinical trial of IT rituximab is needed to further promote clinical application of this therapy. The most common route for IT chemotherapy is lumbar puncture; in this study, 18 of the 27 patients received intra-CSF chemotherapy via lumbar puncture, and the remaining 9 patients were treated using an Ommaya reservoir. There were no treatment-related side effects in either group. The outcome of LM patients is determined by the status of systemic and central nervous system disorders. However, the use of systemic glucocorticoids is useful to treat rapidly progressive lymphoma, which is lysed by direct tumor cells and reduces edema. Steroid therapy can improve patient’s clinical conditions and quality of life. There are few studies of systematic chemotherapy for patients with LM or even secondary central nervous system lymphoma (SCNSL), the majority of treatment options follow the clinical studies of patients with primary central nervous system lymphoma (PCNSL) [13]. A retrospective study performed by IPCG confirmed that systemic HD-MTX is independently associated with better outcome in cases of isolated CNS relapse of aggressive lymphomas [21]. Bokstein et al. [22] reported that in 23 patients with systemic NHL and CNS relapse, HD-MTX was administered intrathecally with Ara-C or oral procarbazine and whole-brain radiation (WBRT) was used in the responders, the median OS was 6 months, 2-year OS was 15%. These results suggested that HD-MTX alone is not enough for SCNSL. A multicenter clinical study performed by Ferreri [23] has shown that in the patients aged 75 years and below with primary CNS lymphoma, the addition of high-dose cytarabine to high-dose methotrexate provides improved outcome with acceptable toxicity as compared with high-dose methotrexate alone. Ferreri et al. [24] proposed a study of PCNSL patients treated with MATILDE regimen (a HD-MTX based polychemotherapy, with thiotepa, idarubicin and Ara-C), followed by response-tailored WBRT, which, at a median follow-up of 12 years, 5-year overall survival rate of 30 ± 7%. The clinical studies conducted by Gregory [25] have shown the addition of rituximab to high-dose methotrexate-based chemotherapy in patients with aggressive B cell CNS lymphoma was associated with improved overall survival.

In cases with NHL-associated CNS complications, LM is more common than parenchymal involvement, although many patients can concurrently experience both types of complications. In our study, patients with parenchymal involvement had better prognoses than other patients. All of these patients were treated with radiotherapy and chemotherapy that included both high-dose systemic MTX (3 g/m^2^) and IT MTX or Ara-C. The systemic chemotherapy regimen for a patient with LM depends on the patient’s general medical status and history of prior treatment as well as the extent of systemic disease. These factors help to guide treatment modalities and may impact survival.

Radiotherapy can alleviate symptoms of LM, treat bulky radiographic disease, and reduce CSF flow abnormalities. Compared with the best supportive care, radiotherapy alone has not been shown to prolong OS for solid tumors [26, 27]. Interestingly, 6 out of 10 patients with parenchymal involvement received cranial and spinal radiotherapy in our study, and all 6 patients exhibited long survival durations. These patients always with obviously symptom, and diagnosed earlier than patients with LM. The diagnoses earlier, the physical condition better. These results suggest that prolonged survival depends on both KPS performance and comprehensive treatment.

For NHL patients with LM, prolonged OS was associated with being near the median age, an early diagnosis of asymptomatic LM, a low IPI score, a good KPS score, and concurrent parenchymal involvement. This study has limitations, including a retrospective design and the inclusion of a small number of patients. Patients with prolonged survival may represent a highly selected cohort, and certain LM patients with poor performance may have been lost to follow-up. Nonetheless, our study revealed characteristics of NHL patients with LM, and it appears that these patients could benefit from multimodal therapy.

## Conclusion

LM is a devastating disease; our study has shown that age, extranodal involvement, IPI, performance status, and parenchymal involvement were predictive factors of prognosis for non-Hodgkin’s lymphoma patients with LM. NHL patients complicated with LM and parenchymal involvement received radiotherapy and had a good prognosis, which benefited from multimodal therapy.

## Electronic supplementary material

Below is the link to the electronic supplementary material.

Supplementary material 1 (DOCX 14 kb)

## Abbreviations

NHL: Non-Hodgkin’s lymphoma
DLBCL: Diffuse large B-cell lymphoma
LM: Leptomeningeal metastases
CNS: Central nervous system
CSF: Cerebrospinal fluid
MTX: Methotrexate
Ara-C: Cytarabine
IT: Intrathecal
MRI: Magnetic resonance imaging
OS: Overall survival
IPI: International Prognostic Index
KPS: Karnofsky performance status
PCNSL: Primary central nervous system lymphoma
SCNSL: Secondary central nervous system lymphoma
