# Supplementary material for: Characteristics and outcomes of non-Hodgkin’s lymphoma patients with leptomeningeal metastases
Source: Int J Clin Oncol. 2018 Mar 20;23(4):783–9. doi: 10.1007/s10147-018-1268-5 (PMC6097078; doi:10.1007/s10147-018-1268-5)
Supplement: Supplementary file 1 — Supplementary material 1 (DOCX 14 kb) [file 10147_2018_1268_MOESM1_ESM.docx]

Supplementary Table 1. Distribution of lymphoma in Tianjin Medical University Cancer Institute and Hospital from 2013 to 2016

|  | No. of patients | percent |
| --- | --- | --- |
| Total | 2784 |  |
| B subtypes | 1801 | 65% |
| Diffuse large B cell lymphoma | 900 | 32% |
| Follicular lymphoma | 288 | 10% |
| Mantle cell lymphoma | 72 | 3% |
| Marginal zone lymphoma | 181 | 7% |
| Chronic lymphocytic leukemia/small lymphocytic lymphoma | 54 | 2% |
| Other disorders of B cell lymphoma | 306 | 11% |
| T/NK subtypes | 685 | 24% |
| Peripheral T-cell lymphoma, not otherwise specified | 177 | 6% |
| Angioimmunoblastic T-cell lymphoma | 98 | 4% |
| Adult T-cell leukemia/lymphoma | 68 | 2% |
| Extranodal NK/T cell lymphoma, nasal type | 95 | 3% |
| Anaplastic large cell lymphoma | 55 | 2% |
| Other disorders of T cell lymphoma | 192 | 7% |
| Hodgkin’s lymphoma | 272 | 10% |
| Nodular lymphocyte predominant Hodgkin’s lymphoma | 8 | 0% |
| Classical Hodgkin’s lymphoma, lymphocyte rich | 41 | 1% |
| Classical Hodgkin’s lymphoma, nodular sclerosis | 103 | 4% |
| Classical Hodgkin’s lymphoma, mixed cellularity | 70 | 3% |
| Classical Hodgkin’s lymphoma, not otherwise specified | 50 | 2% |
| Others | 26 | 1% |

Supplementary Table 2. Distribution of primary extranodal lymphoma in Tianjin Medical University Cancer Institute and Hospital from 2013 to 2016

|  | No. of patients | Percent |
| --- | --- | --- |
| Extranodal sites | 415 |  |
| Nasal sinus/ nasal cavity | 50 | 12% |
| Lung | 7 | 2% |
| Liver | 12 | 3% |
| Tesits | 12 | 3% |
| Intestinal | 33 | 8% |
| Ovary | 2 | 0% |
| Brain | 16 | 4% |
| Tongue | 3 | 1% |
| Stomach | 84 | 20% |
| Bone and soft tissue | 18 | 4% |
| Breast | 19 | 5% |
| Thyroid | 4 | 1% |
| Others | 155 | 37% |

Supplementary Table 3. Survival-related prognostic factors

| Factor | *P* | Relative risk | 95%CI |
| --- | --- | --- | --- |
| Age | 0.8 | 0.98 | 0.841-1.143 |
| IPI (0-1) | 0.045 |  |  |
| IPI (2-3) | 0.719 | 2.928 | 0.008-1012.400 |
| IPI (4-5) | 0.227 | 42.309 | 0.098-18319.718 |
| Parenchyma involvement | 0.061 | 0.152 | 0.021-1.088 |
| Radiotherapy therapy | 0.034 | 0.120 | 0.017-0.853 |
| Histopathology |  |  |  |
| DLBCL | 0.079 |  |  |
| PTCL | 0.032 | 0.001 | 0.000-0.560 |
| Burkitt’s lymphoma | 0.156 | 0.015 | 0.000-4.943 |
| MCL | 0.528 | 0.091 | 0.000-156.9 |
